# Assembled human microbiome and metabolome in chronic kidney disease: Dysbiosis a double-edged sword interlinking Circ-YAP1, Circ-APOE & Circ-SLC8A1

**DOI:** 10.1016/j.toxrep.2025.102058

**Published:** 2025-05-29

**Authors:** Eman A. Zaki, Sherif M. Afifi, Naglaa M. Ammar, Mai O. Kadry

**Affiliations:** a Chemical and Clinical Pathology Department, Faculty of Medicine, Cairo University, Egypt; b Department for Life Quality Studies, Rimini Campus, University of Bologna, Corso d’Augusto 237, Rimini 47921, Italy; c Therapeutic Chemistry Department, National Research Centre, Dokki, Giza 12066, Egypt

**Keywords:** Chronic kidney disease, Metabolomics, Microbiota, Uremic toxins

## Abstract

Dysbiosis is an alteration in microbiota diversity previously elucidated in patients with chronic kidney disease (CKD). Relationship between dysbiosis and CKD is bidirectional; Uremic milieu disturbs the human microbiota on the other hand, gut metabolites influence CKD development. As a result, we outline the possible contribution of microbiota in the pathophysiology, diagnosis and monitoring of CKD. A growing body of research indicates that changes in circular RNAs (circ-RNAs) were observed in CKD with pathogenic implications, including modifying intracellular signaling, exaggerating oxidative stress, cellular apoptosis and inflammation. Additionally, Circ-RNAs exhibit promising role in clinical settings for monitoring, diagnosis, prognostication, and treatment of CKD. Herein blood samples were collected from 60 Egyptian patients with CKD as well as 60 healthy volunteers who served as controls. Following clinical evaluations, OPLS-DA and PCA GC-MS analysis were performed to detect metabolite perturbations. The levels of toxic uremic metabolites, such as urea, hexanedioic acid, ribonic acid, dodecanoic acid, pyrimidine, 1H-indole, 1H-indole-3-acetic acid, butanoic acid, L-cystine, and benzaldehyde linked to renal fibrosis were found to be elevated. Conversely, Reno-protective metabolites, such as short-chain fatty acids; 1H-indole were found to be negatively correlated with indole propionic acid, acetic acid, 2-propenoic acid, tryptophan, tyrosine, and glucitol (AUC 0.65) derived from the gut flora. CKD patients clarified an alteration both gene and protein expression of circRNAs (Circ-YAP1, circ-APOE, and circ-SLC8A1)/mTOR. Moreover, these biomarkers had a significant correlation with clinical investigations such as Creatinine, Glomerular filtration rate (GFR) and albumin/Creatinine (A/C) ratio. These results shed some light on the metabolic biomarkers that are associated with CKD and novel insights into metabolomics/microbiota/Circ-YAP1/circ-APOE/circ-SLC8A1/mTOR interlinked with disease prognosis/diagnosis that could be translated into clinically relevance.

## Highlights

• Chronic kidney disease patients were selected from Al Kasr El Ani Hospital.
• CKD was confirmed in patients via Creatinine, albumin/Creatinine ratio and GFR analysis.
• Observing novel Cir-RNAS (Cir-Yap-1, Cir-APOE and Cir-SCLA3) in CKD patients.
• Reporting alteration in autophagy biomarker mTOR in CKD patients.
• Monitoring Metabolomics biomarkers and correlating them to Microbiota in CKD patients.

## Graphical Abstract

**Figure:**
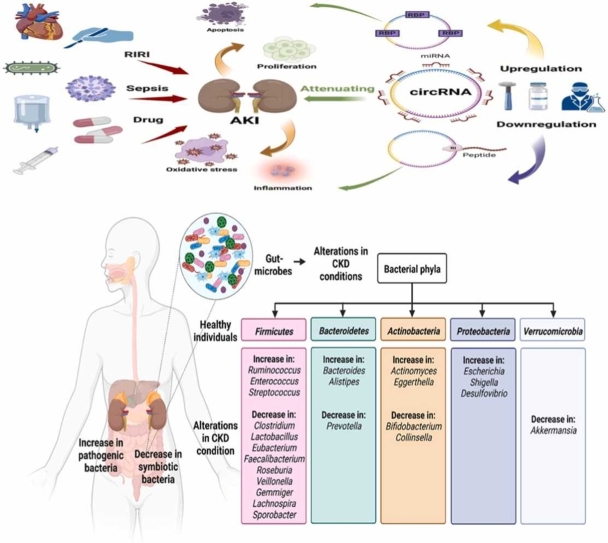

## 1 Introduction

CKD manifests a wide range of pathophysiologic processes linked to impaired kidney function and the progressive decline in glomerular filtration rate (GFR) [50]. The majority of CKD etiologies in children are caused by kidney congenital abnormalities, whereas hypertension and diabetes are the main causes of CKD in adults [6].

In this context, a wide evidence of research has concentrated on the genetic and epigenetic landscape that underlies CKD clinical presentations [46]. Numerous diseases have been linked to non-coding RNAs (ncRNAs) that alter the expression of target mRNAs, and epigenetics is becoming more and more recognized in the pathophysiology of human disease and holds promise for both diagnostic and therapeutic applications. In the field of CKD, a lot of research has concentrated on circular RNAs (circRNAs), which may exert epigenetic modulation upstream of their target miRNAs and lncRNAs. CircRNAs have been connected to the pathophysiology of cancer, neurodegenerative diseases, and autoimmune diseases, highlighting their importance and impact on various body systems. Thus, the primary focus of this article was the evidence regarding circRNAs (Circ-YAP1, Circ-APOE, and Circ-SLC8A1) and the metabolomics approach in different CKD patients. CircRNAs are typically single-stranded, covalently closed RNA molecules [14]**.** CircRNAs can affect transcription, mRNA turnover, and translation through the sponging of RNA-binding proteins and miRNAs, they can also control genes expression. CircRNAs up regulate the mRNAs that miRNAs target and act as miRNA sponges. Through the formation of an RNA-DNA hybrid R-loop structure, they can bind to their target gene at its synthesis locus and act as transcription regulators, causing transcriptional inhibition. CircRNAs can interact with different proteins by forming scaffolds that aids the formation of protein complexes or by acting as protein sponges [52].

CircRNAs were discovered in exosomes released into the blood or urine just like other ncRNAs. On the other hand, CKD patients and HK-2 cells exposed to ischemia-reperfusion injury conditions showed down-regulated circ-YAP1 level [51].

The inflammatory response of CKD is believed to be influenced by gut dysbiosis, which also play an important role in many inflammatory-related diseases [2]**.**

The role of gut dysbiosis in endothelial dysfunction, the vasoconstrictor response, and the subsequent development of hypertension is another way that it may contribute to the progression of CKD [12]. Hypertension and kidney disorders are linked to a decreased number of Lactobacillus species in the gut. Through a sequence of immune response modifications, blood pressure changes, metabolic changes, and chronic inflammation, changes in the gut microbiota may be the initial cause of CKD. Microbial metabolites linked to CKD are generally divided into two categories: reno-protective and harmful metabolites. The impact of trimethylamine N-oxide (TAMO) metabolite on the kidney was reported in a number of human and animal studies including kidney interstitial fibrosis, decline in eGFR, endothelial dysfunction and an elevated risk of cardiovascular disease [16]. The buildup of butanoic acid and 1H-indol acetic acid has been linked to an elevated risk of death and morbidity in CKD patients. Smad signaling, tryptophan metabolism, and tyrosine pathways are all impacted by TAMO, indoxyl sulfate, and p-cresyl sulfate, respectively [9]. Gut-derived metabolites play a dual role in the pathophysiology of CKD; (A) beneficial bacteria generate reno-protective metabolites that prevent kidney damage, while (B) unfavorable bacteria generate harmful metabolites that worsen kidney damage and accelerate the progression of CKD. Short-chain fatty acids (SCFAs), bile acids, indoxyl sulfate, p-cresyl sulfate (PSC), TMAO, and indole derivatives (IDs) are beneficial metabolites.

In the biological samples of patients with CKD, a variety of uremic toxins and other microbial metabolites build up. There have been reports of gut-derived metabolite accumulation and microbial dysbiosis in CKD patients [55]. Changes in the gut microbiota composition following probiotic and prebiotic treatment improved disease outcomes and decreased uremic toxin levels, according clinical trials conducted in CKD patients. Low serum level of uremic toxins, less inflammatory environment, and better renal function were observed in patients with high abundances of Bifidobacterium and Lactobacillus [31].

The molecular phenotype of a biological entity, which represents the "realized" genome, is explained by metabolomics. Because it looks at a person's molecular phenotype, metabolomics is the most promising of the omics sciences in terms of clinical application in comprehensive diagnoses of human health. By using metabolomics analysis to look at the byproducts of nearly all biochemical reactions in the body and the effects of environmental stimuli, it is possible to identify any changes in the human body that are connected to any pathological processes that occur in the human body [32]. By using pattern recognition techniques, it is possible to accurately confirm the disease in a suspected patient by basing a diagnostic method solely on metabolic fingerprinting. Since each organ can be assimilated to highly specific metabolic engines, metabolome analysis can reveal a malfunction in any personal organ [8] or influenced mainly by gut microbiota represents the most individual metabolic variability, and makes each person’s metabotype unique. Tracking metabotypes could allow the evolution of health status and will provide good information on disease onset [3].

The current study focus on the evidence that circRNAs (Circ-YAP1, Circ-APOE & Circ-SLC8A1) and microbiota metabolomics approach detected in different patients of CKD could be of beneficial application in the diagnosis and prognostication of CKD.

## 2 Materials and methods

### 2.1 Experimental section

#### 2.1.1 Subject recruitments

Samples of CKD 60 patients were gathered from the routine laboratory workup; post a systematic appointment to the department of medicine’s clinic at El-KASR El Ainy Hospital (Cairo, Egypt). The study protocol was approved by the Research Ethics Committee, Faculty of Medicine, Cairo University (REC-FOPCU) and obeyed the ethical guidelines of the 1975 Helsinki Declaration. A written informed consent was obtained from each participant before testing. The study was reviewed and approved by the Research Ethics Committees in National research Centre in Cairo (Approval no: 19–013). Patients were divided into two groups; Healthy (n = 60) and CKD (n = 60). In KAH, 45 patients performed hemodialysis and 15 performed peritoneal dialysis. Patients in CKD group had different stages of CKD with a mean GFR of 42.5 ml/min and Creatinine ranging from 2 to 2.5 mg/dl, meanwhile, albumin/creatinine ratio ranging from 23 to 342. A total of 60 healthy subjects with no medical history were enrolled from KAH and served as controls. The healthy control subjects were selected from a biobank, built for our clinical metabolomics program, using an in-house developed algorithm for statistically matching the target disease cohort from Age, gender and others. The demographic and clinical characteristics of the study groups are presented in Table 2.

### 2.2 Study participants

The patient presented to Al-Kasr Al-Ainy hospital Faculty of Medicine, Cairo University, with symptoms of Kidney disease patients; (n = 60) were included in this study. Volunteer healthy persons (n = 60) were participated in the study. Participants were classified according to inclusion criteria into two groups, group A, which included kidney patients, and Group B subjects were healthy controls (HC, n = 60) defined as those who were negative for any symptoms of kidney disease. Approximately 3 ml of blood was collected from each participant in a tube containing 10 % EDTA as an anticoagulant, as described in our previous study [10]. Blood sera were separated from the whole blood samples by centrifuging at 3500 g for 10 minutes and stored at −80°C until further use.

### 2.3 Blood samples analysis

The bio-specimen were examined and analyzed in accordance with the general procedure flow as follows: 1. Gathering and aliquoting samples 2. Quenching and extracting metabolites 3. Pellet re-suspension and sample drying 4. Separation of chromatography and mass spectrometry (GC-MS) 5. Data processing, analysis and identification of metabolites 6. Verification of the findings.

### 2.4 Sample preparation for GC-MS analysis

Blood samples thawed at room temperature for about 5 min and vortexed for 1 min. Two hundred microliters of a ternary mixture of chloroform: methanol: water (2:5:2 % v/v/v) were added to 200 μl of the sample. Then, 200 μl of HPLC-grade methanol was added, mixed thoroughly, and vortexed for 30 s. The mixtures sonicated at room temperature for about 30 min. The samples were then transferred for centrifugation at 1700 ×g at 4 °C. 200 μl of the sample was transferred into screw top vial (2 ml). The samples were then evaporated to dryness under a stream of nitrogen gas. Subsequently, 100 μl of methoxyl amine HCL in pyridine (20 mg/ml) was added and the mixture was left for 2 h at 70 °C. Then, N, O-bis (trimethylsilyl) trifluoroacetamide (BSTFA) with 1 % trimethylsilyl (TMCS) was added to the vial, and the mixture was allowed to react for 1 h at 40 °C [27].

### 2.5 Condition of GC-MS

The GC–MS analysis of the samples was performed using Thermo Gas Chromatograph linked to a MS (Turbomass). An aliquot (1 μl) of the given derivatized sample was injected into PerkinElmer Clarus system having Turbomass software with Elite 5MS 30.0 m × 0.25 mm. internal diameter capillary column containing fused silica as stationary phase with a thickness of 0.25 μm (PerkinElmer). The injector temperature was set at 270 °C. Helium was used as the carrier gas [27].

### 2.6 Temperature program of capillary column

The GC–MS system was initiated with an initial oven temperature of 40 °C, increasing to 200 °C at a heating rate of 5 °C and then to 300 °C at a heating rate of 5 °C. Each process was carried out for about 2 min. The temperatures of the injector and interface were controlled at 220 and 240 °C, respectively. Helium gas was used as a mobile phase, which was delivered at a flow rate of 1.0 ml/min. The MS detection carried out in electron ionization mode through scanning at 50–600 *m/z*. The multiplier voltage and electron energy were set at 323 V and 70 eV, respectively. Solvent delay was 6.5 min, which was due to pyridine, and unknown compounds identified by comparison of the spectra with that of the National Institute of Standards and Technology (NIST) library (2005) and Wiley library (2006). The total time required for the analysis of a single sample was 58 min [27].

### 2.7 Measured biochemical parameters

#### 2.7.1 Renal function biomarkers

##### 2.7.1.1 Serum Creatinine and albumin/Creatinine ratio

Using kits from the Randox Company, the Creatinine and albumin were assessed by measuring the produced colored product, spectrophotometerically at 450 nm (Kadry and Abdel-Megeed, 2023a).

##### 2.7.1.2 Serum Glomerular filtration rate

GFR was assessed using kits that the Randox Company supplied spectrophotometry at 505 nm [25].

##### 2.7.1.3 ELISA determination of serum mTOR, Circ-YAP1, Circ-APOE & Circ-SLC8A1

Using an enzyme-linked immunosorbent assay kit (R&D Systems, MN, USA) in accordance with the manufacturer's instructions, the activities of mTOR, Circ-YAP1, Circ-APOE & Circ-SLC8A1 were determined. After that, a quantitative sandwich enzyme immunoassay was used to assess the experiment. Next, the samples were added to the antibodies pre-coated microplate. Subsequently, the mTOR, Circ-YAP1, Circ-APOE & Circ-SLC8A1 -specific enzyme-linked secondary antibody was added, which was followed by the immobilized antibody. At 450 nm, the absorbance was then determined. The Agient BioTek Microplate reader, Neo2, was used to measure the color intensity at 450 nm [27], [23], [20].

##### 2.7.1.4 Circular RNA Circ-YAP1, Circ-APOE & Circ-SLC8A1 analysis

Circ-RNA was extracted utilizing nucleic acid extraction kit (NucleoSpin® REF. 740901.250) purchased from Macherey- Nagel GmbH & Co. KG- Germany. Serum lysis occurs via equal volume of RA1 buffer and β-mercaptoethanol. Reducing the viscosity and clearing the lysate filtration was done using NucleoSpin®Filter (violet ring) was placed in collection tube then 70 % ethanol was added to the homogenized lysate. Further, the NucleoSpin® RNA Column (light blue ring) was placed in a collection tube then the lysate was loaded to the column and centrifuged. 350 µl of membrane desalting buffer (MDB) was added and centrifuged to dry the membrane then further RA3 buffer was added [21]. NucleoSpin® RNA Column was placed into a nuclease free collection tube and RNA was eluted in 60 µl RNase–free H_2_O. The purity (*A*260/*A*280 ratio) and the concentration of Circ-RNA were determined using spectrophotometry (dual wave length Beckman, Spectrophotometer, USA). Then Quantitative real time –polymerase chain reaction (qRT-PCR) was assessed using kits provided from Bioline, a median life science company, UK (SensiFAST™ SYBR® Hi-ROX One-Step Kit, catalog no.PI-50217 V): Primer sequence for the studied target genes Circ-YAP1, Circ-APOE & Circ-SLC8A1 and reference housekeeping gene *(GAPDH*) were shown in Table 1.

**Table 1:** Primers sequence for Circ-YAP1, Circ-APOE and Circ-SLC8A1.

Gene symbol	Primer sequence from 5′- 3′
Circ-YAP1	F: TAGCCCTGCGTAGCCAGCCAGTTA R: TCATGCTTAGTCCACTGTCTGT
Circ-APOE	F: TGTAAAACGACGGCCAGT R: CAGGAAACAGCTATGACC
Circ-SLC8A1	F: CTTGTGGAGAGCTCGAATTCCAG R: CTCTTCCTCTTTGCTGGTCAGTGG
GAPDH	F: GAAAGCCTGCCGGTGACTAA R: GCGCCCAATACGACCAAATC

### 2.8 Statistical analysis

PCA, and OPLS-DA were used to analyse differences between the groups. Two-tailed Student’s t-tests were performed *via* SPSS 17.0 software (SPSS Inc, Chicago, IL, USA). Compounds with variable importance in projection (VIP) values > 1.0 in OPLS-DA and two-tailed student’s *t*-test p values < 0.05 were deemed potential discriminant metabolites using Venn plot. For univariate analysis, Volcano analysis and fold change (FC) were performed using MetaboAnalyst 6.0 to evaluate statistical significance (http://www. metaboanalyst.ca). Receiver operating characteristic (ROC) curves were conducted to evaluate the diagnostic efficacy of candidate metabolic biomarkers (GraphPad Prism 6.0, San Diego, CA, USA &SPSS 17.0). The optimal cut-off point used to classify diseased cases from non-diseased was determined by the Youden index (sensitivity + specificity - 1), which was considered a common summary measure of the ROC curve. Binary logistic regression was also performed to select the best combination of follicular biomarkers using SPSS 17.0 software. (SPSS Inc., USA). Furthermore, a heat map and pathway analysis was performed to provide an overview of significantly changed metabolites through MetaboAnalyst 6.0. (http://www.metaboanalyst.ca) and the Kyoto Encyclopedia of Genes and Genomes (KEGG; http://www.kegg.jp) pathways with raw p values < 0.05 and impact values > 0 were deemed to be potentially related biomarkers.

## 3 Results

### 3.1 Modulation of kidney function tests

In a study containing about 42 males and 18 female CKD patients within the age of 66 years CKD patients elucidated a significant increase in kidney function tests including Creatinine, Albumin/Creatinine (A/C) ratio and a decrease in Glomerular filtration rate (GFR) with a mean value of (2.54 mg/dl, 81.25 and 42.3 ml/min/1.73 m^2^) respectively, and in G3b stage ranging from (30−44) Moderately to severely decreased kidney function as compared to the healthy subjects (42 males and 18 females within the age of 65 years) with Creatinine, Albumin/Creatinine (A/C) ratio and Glomerular filtration rate (GFR) with a mean value of (0.68 mg/dl, 9.93 and 101.6 ml/min/1.73 m^2^) respectively and in G1 ≥ 90 (Normal) as represented in (Table 2).

**Table 2:** Clinical characteristics and Kidney function tests including Creatinine (CRT), Glomerular filtration rate (GFR) and Albumin/Creatinine ratio (A/C) in chronic kidney disease patients and healthy subjects.

	Age (years)	CRT (mg/dl)	GFR (ml/min/1.73 m^2^)	A/C ratio	Stage	Gender (Male/Female)
CKD (n = 60)	66 ± 1.11^a^	2.54 ± 0.2^b^	42.3 ± 5.2^b^	81.25 ± 7.5^b^	G3b 30–44 (Moderately to severely decreased)	42/18 (No.)
Control (n = 60)	64.7 ± 0.9^a^	0.63 ± 5.8^a^	103.6 ± 2.8^a^	9.14 ± 1.28^a^	G1 ≥ 90 (Normal)	42/18 (No.)

Values are expressed as mean ± SEM, n = 60. Data were analyzed using independent t- test, P value ˂0.05. Different letters indicating significant differences at p < 0.05.

### 3.2 Multivariate metabolic analysis

**Fig. 1:**
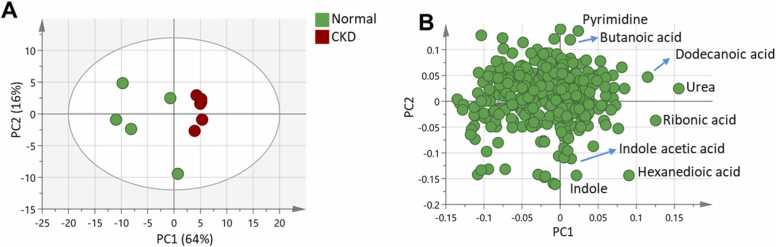
**(A)** PCA score plot shows distinct separation between the CKD group and healthy control along the first principal component, accounting for significant metabolic changes. (B) PCA loading plot identifies abundant metabolites in CKD group. The identified metabolites in CKD group were as follow: urea, hexanedioic acid, ribonic acid, dodecanoic acid, pyrimidine, indole, indole acetic acid, and butanoic acid.

**Fig. 2:**
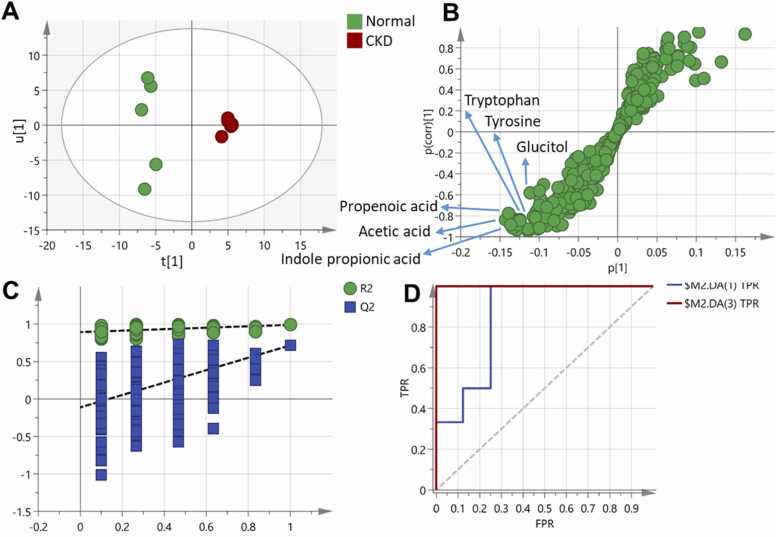
**(A)** Score plot of the OPLS-DA model showing clear separation between the CKD group and the healthy control group along the predictive component, indicating significant metabolic differences. (B) S-plot highlighting the metabolites driving the separation between the groups, including increased levels of indole propionic acid, acetic acid, propenoic acid, tryptophan, tyrosine and glucitol that are derived from the gut flora in healthy control group while same metabolites outlined by PCA were also elevated in the CKD group. (C) Permutation test validating the model's reliability, with a negative intercept supporting the robustness of the OPLS-DA model. (D) ROC curve of the OPLS-DA model demonstrating excellent diagnostic performance with an AUC of 0.65 for distinguishing CKD group from healthy control group.

Multivariate data analysis revealed specific metabolic signatures associated with CKD and the healthy control group. A total of 78 metabolites (Table 3) were detected and survived quality control filtering with RSD< 30 %. Unsupervised PCA was performed to visualize the global variation in the data. The PCA score plot showed a clear separation between the CKD and the healthy control group along the first principal component, indicating significant metabolic perturbations in the CKD group relative to the control with the first two components explaining 80 % of the variation (Fig. 1A). The loading plot identified the metabolites that increased in the CKD group such as urea, hexanedioic acid, ribonic acid, dodecanoic acid, pyrimidine, indole, indole acetic acid, butanoic acid, cystine, and benzaldehyde, that are associated with renal fibrosis, endothelial dysfunction and decline in the eGFR (Fig. 1B). Consequently, OPLS-DA model was applied to identify the metabolites that were significantly altered in the CKD group compared to the control group. The OPLS-DA model (Fig. 2) compared CKD vs control had a satisfactory R2Y and Q2 of 0.9 and 0.91 respectively. A clear separation between the two groups was observed in the scores plot along the predictive component, indicating substantial metabolic differences (Fig. 2A). The metabolites that contributed most to the separation were identified based on the S plot that modeled the covariance and correlation structures between the X and Y matrices (Fig. 2B). These included increased levels of urea, hexanedioic acid, ribonic acid, dodecanoic acid, pyrimidine, indole, indole acetic acid, butanoic acid, and cystine, in the CKD group compared to control. On the hand, indole propionic acid, tryptophan, tyrosine, glucitol and acetic acid were increased in the control group. The permutation test confirmed the validity of the model with negative intercept value (Fig. 2C). ROC curves were plotted to evaluate the diagnostic potential of the developed OPLS-DA model. The area under the curve for differentiating CKD from Control was 0.89, indicating excellent diagnostic accuracy (Fig. 2**D**). GC-MS metabolic analysis revealed a significant Contrary, Reno-protective metabolites including short-chain fatty acids and microbiota related metabolites can prevent the progression of CKD by suppressing the disruption of the epithelial barrier and regulating the anti-inflammatory response.

**Table 3:** Identified metabolites detected by GC-MS with RSD< 30 %.

No.	Rt (min)	RI	Metabolite name
1	4.06	948	Acetic acid
2	4.16	955	Propanoic acid
3	4.52	978	Hexanoic acid
4	4.68	988	Myrcene
5	5.24	1019	Pyridine
6	5.51	1033	Benzyl Alcohol
7	6.19	1068	Hexanoic acid
8	6.28	1072	methylcatechol
9	6.87	1102	Nonanal
10	6.94	1105	Propenoic acid
11	7.23	1119	Ethanedioic acid
12	7.54	1133	Glycine
13	8.33	1169	Mentha-dienol
14	8.41	1173	Octanoic Acid
15	8.83	1192	Propanedioic acid
16	9.43	1218	Alanine
17	9.62	1227	Valine
18	9.77	1232	Pantoyl lactone
19	9.98	1242	Benzaldehyde
20	9.99	1242	Urea
21	10.16	1250	Benzoic acid
22	10.50	1265	Valerate
23	10.75	1275	Butenedioic acid
24	10.85	1280	leucine
25	10.94	1283	Butanoic acid
26	10.96	1285	Glycerol
27	11.29	1299	Thymol
28	11.31	1300	Isoleucine
29	11.36	1302	Proline
30	11.36	1302	Quinoline
31	11.79	1322	Indene
32	12.25	1343	Pyrimidine
33	12.55	1357	Butyl caprylate
34	12.57	1358	Nonanoic acid
35	13.03	1380	Copaene
36	13.40	1396	Threonine
37	13.57	1405	Pentanedioic acid
38	14.32	1443	Dihydroxybutanoic acid
39	14.36	1444	Pentenedioic acid
40	14.38	1446	Homoserine
41	14.59	1456	Decanoic acid
42	16.00	1527	Methionine
43	16.08	1531	Proline
44	16.12	1532	Aspartic acid
45	16.77	1565	Cysteine
46	17.85	1620	Dodecanoic acid
47	17.91	1624	Ornithine
48	18.02	1631	Glutamine
49	19.11	1698	Ribose
50	19.19	1702	Hexanedioic acid
51	19.22	1703	Tridecanoic acid
52	19.40	1713	Xylitol
53	19.62	1723	Indole
54	20.17	1750	Ribitol
55	20.26	1755	Arabitol
56	20.71	1777	Mannopyranose
57	20.83	1783	Phosphoric acid
58	21.08	1795	Azelaic acid
59	21.26	1805	Ribonic acid
60	21.97	1846	Caffeine
61	22.25	1863	Pentadecanoic acid
62	22.32	1867	Galactose
63	22.65	1887	Tyrosine
64	23.47	1937	Glucitol
65	23.16	1918	Glucose
66	24.00	1970	Indole acetic acid
67	24.04	1972	Galactitol
68	25.00	2032	Gluconic acid
69	25.69	2077	Oleic Acid
70	25.81	2084	Indole propanoic acid
71	26.31	2117	Myo-Inositol
72	26.77	2149	Nonanoic acid
73	27.64	2211	Linolenic acid
74	27.81	2224	Tryptophan
75	29.07	2321	Cystine
76	33.19	2634	Docosanoic acid
77	33.28	2641	Lactose
78	39.23	3150	Cholesterol

### 3.3 Modulation of circular-RNA (circ-YAP1, circ-SLC8A1 and circ-APOE)

**Fig. 3:**
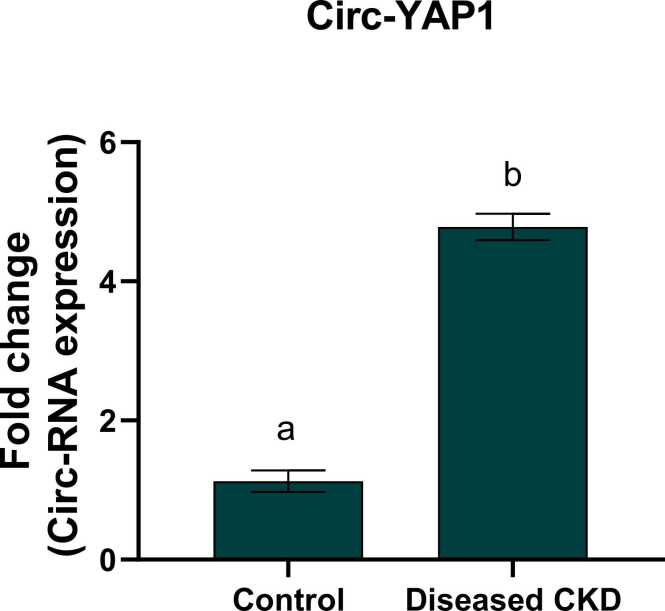
Fold change of circ-YAP1 in control and diseased CKD study groups. Values are expressed as mean ± SEM, n = 60. Data were analyzed using independent t- test, P value ˂0.05. Different letters indicating significant differences at p < 0.05.

**Fig. 4:**
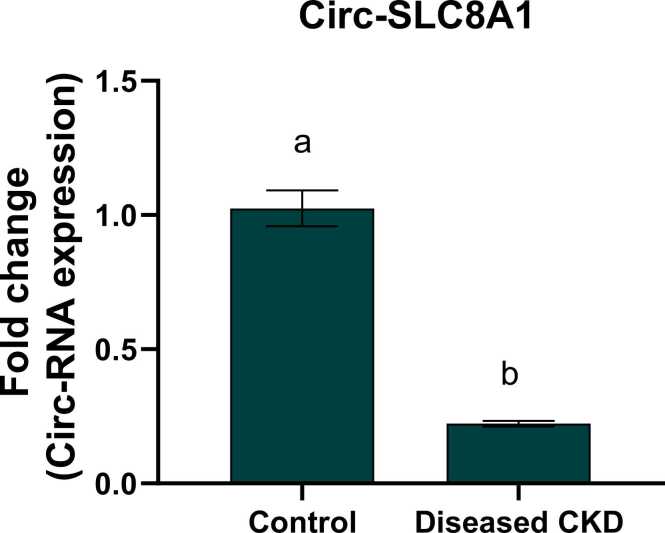
Fold change of circ-SLC8A1 in control and diseased CKD study groups. Values are expressed as mean ± SEM, n = 60. Data were analyzed using independent t- test, P value ˂0.05. Different letters indicating significant differences at p < 0.05.

**Fig. 5:**
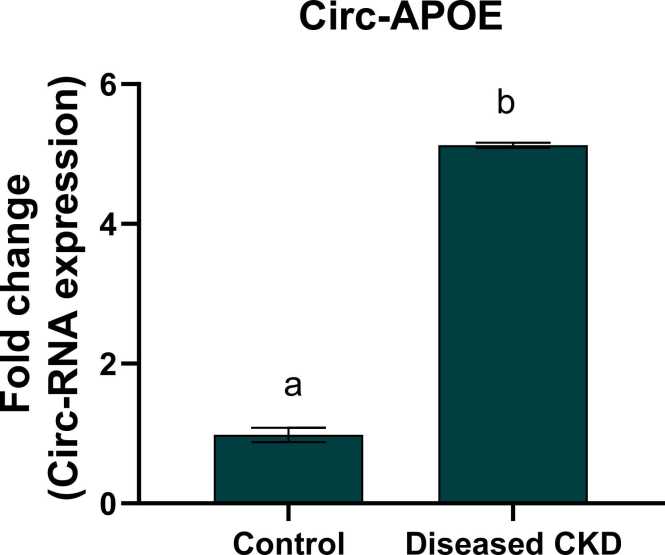
Fold change of circ-APOE in control and diseased CKD study groups. Values are expressed as mean ± SEM, n = 60. Data were analyzed using independent t- test, P value ˂0.05. Different letters indicating significant differences at p < 0.05.

The current study elucidated a remarkable up regulation in circ-YAP1 and circ-APOE gene and protein expression (Table 4) with a fold change (4.78 & 5.1) respectively and a significant down regulation in circ-SLC8A1 expression with a fold change 0.22 in CKD patients as compared to the healthy subjects, reflecting the vital role of circular RNA in the prognosis, diagnosis and progression of CKD (Fig. 3, Fig. 4, Fig. 5).

**Table 4:** Protein expression of YAP1, SLC8A1 and APOE in both CKD and healthy groups.

	Control	Diseased CKD
YAP1(ng/ml)	0.73 ± 0.07^a^	5.68 ± 0.13^b^
SLC8A1 (ng/ml)	4.21 ± 0.09^a^	1.02 ± 0.045^b^
APOE (ng/ml)	16.06 ± 0.27^a^	180.5 ± 2.08^b^

Values are expressed as mean ± SEM, n = 60. Data were analyzed using independent t- test, P value ˂0.05. Different letters indicating significant differences at p < 0.05.

### 3.4 Modulation of autophagy biomarker mTOR

**Fig. 6:**
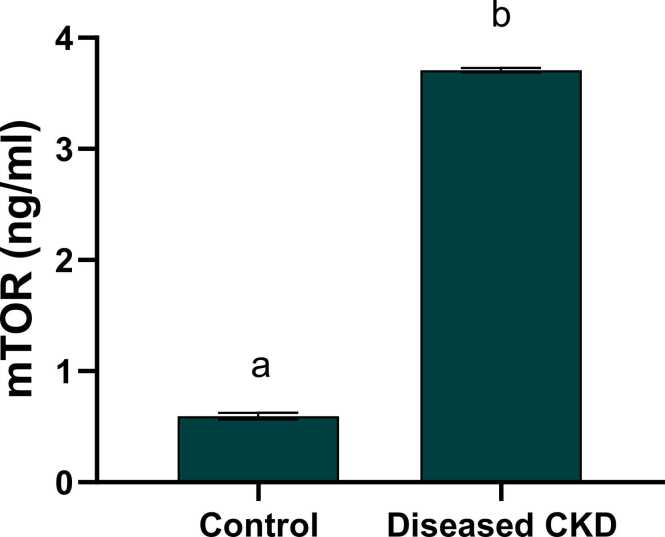
level of mTOR in control and diseased CKD study groups. Values are expressed as mean ± SEM, n = 60. Data were analyzed using independent t- test, P value ˂0.05. Different letters indicating significant differences at p < 0.05.

**Fig. 7:**
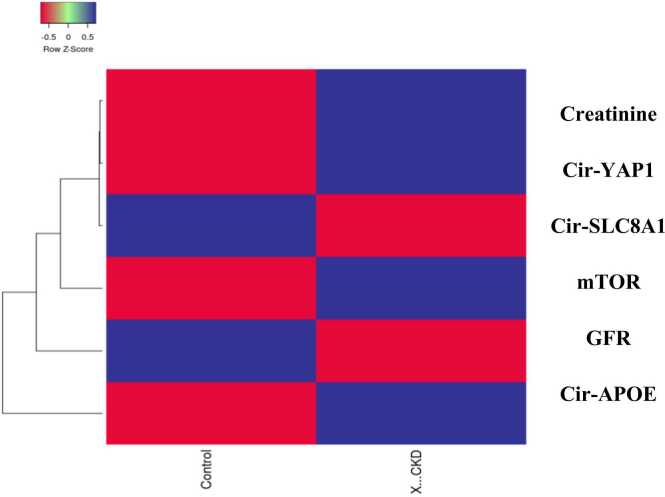
Heatmap representing different gene expressions; Blue represents high score while red represents low score.

The current study elucidated a noticeable elevation in the autophagy biomarker mTOR protein expression in CKD patients as compared to the healthy subjects with a mean value of 3.6 ng/ml reflecting the chief role of autophagy in the incidence of chronic kidney disease and it’s crosslink with Circ-YAP1 (Fig. 6). Fig. 7 illustrated a Heatmap representing different gene expressions.

## 4 Discussion

About 13.4 % of people worldwide suffer from chronic kidney disease, which is a steadily rising healthcare burden [33]. A growing body of evidence indicates that micro-biomes play a significant role in preserving human health. The collection of all microbial DNA found in human body, which is dispersed throughout the body's organs, is known as human microbiome [15]. These microorganisms are essential for vitamin synthesis, immune response stimulation and regulation, digestion and metabolism, and pathogen defense. In healthy conditions, this microbial community and the host have a symbiotic relationship [17]. A disturbance in the microbial balance is known as dysbiosis, and it is linked to a number of pathological conditions, including CKD [56]. However, Firmicutes, Bacteroidetes, Actinobacteria, and Proteobacteria are the most prevalent bacterial phyla in the gut. The gut–kidney axis is sometimes used to describe the interaction between gut bacteria, their metabolites and kidney function. According to recent research, the pathophysiology of CKD with severe CKD outcomes is significantly influenced by aberrant gut microbiota [11]. The human microbiome is primarily found in the gut, which has about ten times as many microbial cells as other human cells. These cells produce a range of metabolites that affect the kidney in addition to controlling the metabolism of nutrients. Dysbiosis and the pathophysiology of CKD are bi-directional [18]. As represented in the present study increasing levels of uremic metabolites, such as urea, pentenedioic acid, hexanedioic acid, ribonic acid, dodecanoic acid, pyrimidine, indole, indole acetic acid, butanoic acid, cystine, and benzaldehyde was linked to renal fibrosis, endothelial dysfunction, decrease in the estimated glomerular filtration rate (eGFR) and higher morbidity in CKD individuals.

Additionally, there is a negative correlation between the progression of CKD and serum levels of tyrosine and tryptophan. However, short-chain fatty acids inhibit the progression of CKD by inhibiting the disruption of the epithelial barrier and controlling the anti-inflammatory response and reno-protective metabolites [7]. Three types of uremic toxins are distinguished: middle molecules, protein-bound solutes, and free water-soluble low-molecular-weight solutes. The development of CKD and its co-morbidities, such as CVD, is linked to the buildup of uremic toxins in the blood and tissues. Uremic toxins indoxyl sulfate, p-cresyl sulfate, hippuric acid, TMAO, ADMA, TNF-α, and IL-6 and their effects on the kidneys and heart were previously addressed. Many protein-bound uremic toxins originate in the gut and are byproducts of intestinal bacteria breaking down aromatic amino acids, such as indoxyl sulfate (IS); indole is a metabolic product of bacterial tryptophanase breaking down dietary tryptophan. After being generated by intestinal bacteria, indole is taken up by the portal circulation and goes to the liver, where cytochrome P450 2E1 hydroxylates it to 3-hydroxy indole, which is then sulfated by sulfotransferase 1A1 to yield IS. Due to its strong protein binding to albumin in the bloodstream, IS is not well removed by dialysis. IS levels rise in the blood as kidney function deteriorates, and this increase aids in the development of CKD [29]. By producing ROS, depleting anti-oxidative systems, and causing inflammation and fibrosis, IS demonstrated nephrotoxic effects. An important factor in the pathological effects of IS on the kidneys is NF-κB. IS-induced NF-κB activation in human proximal tubule cells (HK-2) inhibited cell division, accelerated senescence by inducing p53, and induced fibrosis by inducing the expression of TGF-β1 and PAI-1. It was also suggested that p53 induction plays a role in renal fibrosis by promoting TGF-β1 expression and subsequently activating Smad3. By activating the renin-angiotensin system, IS caused an EMT-like process both in vitro and in vivo, which further exacerbated renal fibrosis. Additionally, IS stimulates the production of monocyte chemoattractant protein (MCP-1), a chemotactic cytokine linked to the recruitment and activation of macrophages in tubulointerstitial inflammation, and intercellular adhesion molecule 1 (ICAM-1), which is linked to monocyte infiltration into the kidney [29].

In CKD patients, the levels of gut flora-derived microbiota metabolites such as indole propionic acid, glucitol, acetic acid, and propenoic acid are negatively correlated with p-cresyl sulfate and indole concentrations [54]. Long-term survival, development of CKD and buildup of uremic toxin were inversely correlated to Bifidobacterium, Lactobacilli and short-chain fatty acid-producing bacteria as Faecalibacterium prausnitzii, Roseburia, and Prevotella [53]. On the other hand, CKD patients' elevated levels of secondary bile acids and uremic toxins are positively correlated to Eggerthella lenta, Fusobacterium nucleatum, Alistipes shahii, Paraprevotella, Pseudobutyrivibrio, and Collinsella stercoris; this finding led to the suggestion that this signature could be used to distinguish between CKD patients and healthy individuals [56]. Previous clinical researches were conducted on the use of probiotics to alter the production of indole products by intestinal bacteria to prevent CKD. Pre-clinical studies have shown that probiotics can lower plasma indole and prevent CKD and atherosclerosis. Nonetheless, human clinical trials have shown the same findings regarding the effectiveness of probiotic supplementation. The administration of either lactofermented Annurca apple puree or Lactobacillus bacteria reduced plasma indole levels in comparison to baseline indole in a clinical trial involving patients with CKD risk factors. Conversely, giving a probiotic supplement containing lactobacilli and bifidobacteri to healthy individuals who consumed a high-fat, high-calorie diet for four weeks had no impact on the levels of indole in their plasma [29].

The buildup of metabolic waste products is linked to CKD; Elevated blood urea and creatinine, along with haematologic electrolyte, endocrine and skeletal problems are typical manifestations of these alterations [34].

Proteins breakdown produces urea, the most prevalent metabolite and the main nitrogenous waste product of metabolism. Since the kidneys get rid of it through urine, measuring its concentration in urine and then blood has clinical manifests in evaluating kidney function. Uremia is a clinical condition marked by fluid overload, electrolyte imbalances, metabolic abnormalities, and physiological changes that are linked to declining renal function, chronic and end-stage renal disease [36]. Numerous illnesses can cause uremia, from systemic disorders that harm the kidneys to primary renal disorders like immunoglobulin A nephropathy, focal segmental glomerulo-sclerosis, membrane-proliferative glomerulonephritis, and polycystic kidney disease [26], [37], [42], [45].

Urine purines and pyrimidines are crucial diagnostic indicators for a number of inborn metabolic errors (IEM). They are also a component of the general metabolic screening for patients with kidney stones, anemia, neurological symptoms and immune deficiencies [44]. Based on the clinical picture, they can also determine whether a urea cycle disorder is likely. Numerous biological processes, including the synthesis of RNA and DNA, as well as the formation of high-energy nucleotides like adenosine triphosphate, depend on purine and pyrimidine nucleotides [39]. Changes in purine and pyrimidine metabolism are linked to polygenic and CKD. Many of these genes code for renal tubule transporters and related proteins. Urinary purines and pyrimidines are difficult to excrete when renal function is compromised [5].

Microbiota metabolites were altered in CKD patients. While uremic toxins, such as indoles, ammonia, and trimethylamine N-oxide, which are produced by the gut microbiota, accumulate due to their overproduction and decreased excretion by impaired kidney function, making CKD more likely to develop on the other hand SCFAs can improve health and were noticed to be reduced. Reduced production of SCFAs leads to impaired CKD because of gut dysbiosis and decreased dietary fiber intake, which both lowers SCFA production and contributes to increased amino nitrogen, which the gut microbiota can convert [35].

Numerous bacterial metabolites are produced by the intestinal microbiota's metabolic activity. Among these, certain bacterial species convert tryptophan into indole and compounds related to indole. Plasma level of tryptophan-related metabolites were elevated in CKD patients and revealed a positive association with tubulointerstitial fibrosis. CKD changed the abundance of some gut microbiota (decrease and increase). Clostridium IV, Turicibacter, Pseudomonas, and Lactobacillales were positively correlated with plasma tryptophan level, while Oscillibacter, Blautia, and Intestinimonas showed a negative correlation [54]. Indole-related compounds are active on the intestinal mucosa and have generally positive effects in various experimental settings, despite initially being involved in the communication of the intestinal microbial community. Following absorption, the liver partially breaks down indole to produce the co-metabolite indoxyl sulfate (IS) [54]. While indole has been shown to have some anti-inflammatory properties for liver cells, indoxyl sulfate is a well-known uremic toxin that damages kidney cells, exacerbating CKD. Endothelial dysfunction is another known effect of IS [48]. CKD patients accumulate more uremic toxins in the form of indole-acetic acid. The development of CKD, the risk of cardiovascular disease, and overall mortality are all linked to gut-derived metabolites indoxyl sulfate.

In CKD patients, cardiovascular morbidity and mortality have been associated with the protein-bound uremic toxin indole-3-acetic acid (IAA). The impact of IAA on cardiovascular alterations in rats with adenine sulfate-induced CKD was previously investigated [22]. Compared to CKD, IAA-exposed CKD rats had higher circulation and decreased excretion of IAA. MDA, β-MHC, TNF-α, IL-1β, and NF-κB levels were found to be significantly raised in CKD groups exposed to IAA [40].

Recently identified as a uremic toxin, phenylacetic acid (PAA) inhibits the expression of inducible nitric oxide synthase (iNOS) and plasma membrane calcium ATPase, potentially contributing to the remodeling of arteries in patients with CKD [41]. By suppressing cell proliferation and cytokine-induced endothelial expression of adhesion molecules and pro-inflammatory cytokines; NO stops atherogenesis and inflammation in vessel walls. Phenylacetic acid was elevated in end-stage renal failure patients and suppresses the expression of iNOS leading to higher cardiovascular morbidity and atherosclerosis [13].

Essential branched chain amino acid and ribonic acid are linked to cardio-renal events, eGFR and decreased risk of the combined renal endpoint in Diabetes mellitus patients. Myo-inositol and ribonic acid were longitudinally linked to a higher risk of eGFR ≥ 30 %, inversely correlated with eGFR≤ 30 % and positively correlated with macro-albuminuria and risk of renal endpoint.

Hydroxy butyrate 3,4-dihydroxybutanoic acid was linked to micro- and macroalbuminuria, urinary albumin excretion rate, and eGFR [49].

In CKD; myo-inositol, dodecanoic acid, N-acetylputrescine, and anthranilic showed a decrease in the genera that produce short-chain fatty acids (Bacteroides, Prevotellaceae_UCG-001, Roseburia, and Lachnospiraceae_NK4A136_group), despite the fact that a significant portion of the metabolomic associations were with low eGFR [8]. A negative correlation was found between renal fibrosis and Bacteroides and Prevotellaceae. A positive correlation was found between CKD progression and Parasutterella and Alistipes. Neural damage was positively connected with myo-inositol, dodecanoic acid, anthranilic acid, and N-acetylputrescine.

Anaerobic bacteria in the intestinal tract produce short-chain fatty acids (SCFAs), which are important for the systemic as well as local intestinal barrier [1]. There are fewer SCFA-producing bacteria in the gut microbiota of patients with renal stones [30].

One of the most prevalent SCFAs, butanoic acid has immune-suppressive or anti-inflammatory properties. Numerous illnesses, including diabetes, cancer, and kidney disease, are associated with dysbiosis of butanoic acid metabolism [4].

Hexanedioic acid members and supporting chemicals showed effects such as kidney histopathological changes, changes in enzyme activities, and kidney/heart lesions. Hexanedioic acid was significantly elevated in CKD patients with lower protein levels [38]. One important step in the pathophysiology of kidney stone formation is cystinuria that occurs by crystallization of L-cystine [20].

It was estimated that the tyrosine/phenylalanine ratios in the groups under study differed significantly. By converting phenylalanine to tyrosine, the kidney primarily helps the body's circulatory system receive tyrosine. As the phenylalanine hydroxylase enzyme activity declines in CKD, there is a notable reduction in tyrosine release, which lowers its serum levels. As a result, the Tyr/Phe concentration ratio is lower in CKD than in healthy people. Protein depletion and impaired synthesis of aromatic amine modulators like dopamine, norepinephrine, or epinephrine can be caused by a tyrosine deficiency [28].

One potential biomarker for renal function is indole propionic acid, a metabolite that protects the kidneys. Patients with CKD and those with declined eGFR have significantly lower levels of indole propionic acid, which is produced by a healthy gut. Endothelial barrier disruption is prevented by gut-derived metabolites (SCFAs). SCFA levels were noticeably lower in renal failure. Children with CKD who have congenital kidney and urinary tract abnormalities demonstrated a decrease in propionate and butyrate plasma levels as the relative abundance of the Verrucomicrobia, Akkermansia, and Bifidobacterium bifidum [24]. Propionate and butyrate plasma levels were higher in CKD children who had an abnormal ambulatory blood pressure monitoring profile [54].

CKD had elevated plasma levels of tryptophan-related metabolites, which were positively correlated with tubule-interstitial fibrosis [47]. The abundance of certain gut microbiota was altered in CKD; Oscillibacter, Blautia, and Intestinimonas exhibited a negative correlation with plasma tryptophan levels, whereas Clostridium IV, Turicibacter, Pseudomonas, and Lactobacillales exhibited a positive correlation (Kadry et al., 2024).

Glucitol level was estimated to be associated with end-stage renal disease, risk factors and latent variable for kidney function (creatinine, cystatin C, β2-microglobulin) [43].

In contrast to healthy subjects, the current study demonstrated a notable up regulation in circ-YAP1, circ-SLC8A1, and circ-APOE expression in CKD patients. By serving as a sponge for miR-21–5p and triggering signal transduction through the PI3K/Akt/mTOR pathway, which are essential for controlling inflammatory and fibrotic reactions in response to renal tissue damage, overexpression of circ-YAP1 reduced Ischemia-reperfusion injury (IRI) in vitro [19]. 4983 circRNAs were identified as differentially expressed in a different study using a unilateral IRI murine kidney model. Previous analysis revealed that the parental genes of these circRNAs were implicated in adhesion junctions, focal adhesion, and the control of actin cytoskeleton pathways. Two hub genes—circ-SLC8A1 (mmu_circ_0000823) and circ-APOE (mmu_circ_0014064)—were linked to numerous differentially expressed miRNAs and mRNAs [55]. Additionally, these circRNAs exhibit aberrant expression in solid organ tumors and CKD, where they were linked to angiogenesis, cell migration, epithelial-to-mesenchymal transition, and the development and spread of renal fibrosis. Thus, it is conceivable that these circRNAs play a critical role in the development of chronic damage and the progression from AKI to CKD [46].

## 5 Conclusion

A significant part of the pathophysiology of many diseases is microbial dysbiosis. We looked into the dual function of the gut microbiota and the products of its metabolism in the development of chronic kidney disease. TAMO, indoxyl sulfate, p-cresyl sulfate, and other toxic microbial metabolites are known to build up in CKD patients, and their levels are known to increase as the disease progresses. Negative outcomes in patients with chronic kidney disease are associated with lower levels of bifidobacterium, lactobacillus, and bile acid composition. The intricate relationship between the gut microbiota and related metabolites may be responsible for the disease's development by coordinating subclinical alterations in the pathophysiology of CKD. In order to determine the gut microbiota community, metabolic pathways, and microbial genes linked to chronic kidney disease (CKD), we advise performing omics-based research such as meta-genomics and meta-transcriptomics, examining the gut microbiota at various stages of the disease, particularly in the early stages. iii. Conducting early dietary intervention studies for patients with chronic kidney disease. iv. Evaluation of CKD patients' blood and urine microbiome investigations. These directions might provide information about the metabolic pathways, etiology, and possible treatments for chronic kidney disease.

## Study limitation

Larger sample size is needed to confirm our findings. GC-MS alone may not capture the full metabolomics landscape, necessitating integration with LC-MS NMR or other Omics approaches for a comprehensive understanding. Also GCMS can’t cover all metabolites of organism and there is a dynamic limitation for one time detection in metabolomics platform.

## Author statement

All authors revised the manuscript and agree for publication

## Author contribution

Eman A. Zaki performed metabolomics procedure and RTPCR and revised the manuscript. Mai O. kadry performed RTPCR, metabolomics procedure and wrote the manuscript. Naglaa M. Ammar performed metabolomics procedure and analysis and revised the manuscript. Sherif M. Afifi performed metabolomics analysis

## Consent of publication

National research center, ethics number 19–302.

## Funding declaration

The current work received NO financial support.

## Declaration of Competing Interest

The authors declare that they have no known competing financial interests or personal relationships that could have appeared to influence the work reported in this paper.

## Data Availability

No data was used for the research described in the article.
